# Pressure Injuries and Management after Spinal Cord Injury

**DOI:** 10.3390/jpm12071130

**Published:** 2022-07-12

**Authors:** Nicole M. Vecin, David R. Gater

**Affiliations:** 1 University of Miami Miller School of Medicine, Miami, FL 33136, USA; n.vecin@med.miami.edu; 2 Department of Physical Medicine and Rehabilitation, University of Miami Miller School of Medicine, Miami, FL 33136, USA; 3 Christine E. Lynn Rehabilitation Center for the Miami Project to Cure Paralysis, Miami, FL 33136, USA; 4 The Miami Project to Cure Paralysis, University of Miami Miller School of Medicine, Miami, FL 33136, USA

**Keywords:** spinal cord injury, tetraplegia, paraplegia, pressure injury, wound management

## Abstract

Spinal cord injury (SCI) results in motor paralysis and sensory loss that places individuals at particularly high risk of pressure injuries. Multiple comorbidities associated with autonomic, cardiovascular, pulmonary, endocrine, gastrointestinal, genitourinary, neurological, and musculoskeletal dysfunction makes it even more likely that pressure injuries will occur. This manuscript will review the structure and function of the integumentary system, and address the multidisciplinary approach required to prevent and manage pressure injuries in this vulnerable population.

## 1. Introduction

In addition to paralysis and sensory loss, persons with SCI have multiple comorbidities, including neurogenic orthostatic hypotension, autonomic dysfunction, neurogenic restrictive and obstructive lung disease, neuropathic pain, spasticity, neurogenic bladder, neurogenic bowel, neurogenic obesity, sarcopenia, and metabolic syndrome (including diabetes mellitus, hypertension, dyslipidemia, and systemic inflammation) that increases their risk of pressure injuries. While early education about pressure-injury prevention, including appropriate support surfaces, wheelchair and seating systems, frequent repositioning, optimal transfer techniques, nutrition, physical activity, weight management, and smoking cessation may provide the best strategy for pressure-injury treatment in the form of prophylaxis [1,2], once a pressure injury occurs, early wound care is essential to promote timely wound-healing [3]. Management requires multidisciplinary intervention to determine and fix the cause of the pressure injury, optimize psychosocial support, provide complete pressure relief of the wound, optimize nutrition, minimize wound bioburden, optimize support surfaces and wheelchair seating systems, eradicate infections, and if necessary, provide surgical intervention with myofasciocutaneous flaps and appropriate post-operative care. Comorbidities of infection, sepsis, autonomic dysreflexia and spasticity makes these wounds especially dangerous to persons with SCI, and societal cost of managing these wounds may exceed USD $11 billion annually [1,2].

## 2. Normal Tissue over Bony Prominences

Tissue overlying bony prominences is comprised of the skin, hair, sweat glands, fascia, blood vessels, nerves, and muscle. The major components of the skin include (superficial to deep) the epidermis, dermis, skin appendages, and subcutaneous fat, and their primary role is to serve as a barrier to maintain internal homeostasis [4]. There are five layers of the epidermis in descending order, which includes the stratum corneum, stratum granulosum, stratum spinosum, basal cells, and rete ridges. The *stratum corneum* contains dead cells that are large, flat, plate-like envelopes filled with keratin that provide a major physical barrier against environmental impacts. On most body surfaces, the stratum corneum is 10–25 layers thick, but the palms and soles have as many as 100 layers. The *stratum granulosum* is comprised of keratinocytes that contain dark keratohyalin granules. Keratohyalin contains two proteins, profilaggrin (the precursor to filaggrin) and involucre, that play a role in the formation of the cell envelope of stratum corneum cells. Keratinocytes also contain lamellar granules, comprised of polysaccharides, glycoproteins, and lipids, that extrude into the intercellular space and act as the “cement” that holds stratum corneum cells together. The stratum spinosum is composed of keratinocytes connected by desmosomes that appear as spines or intercellular bridges extending between keratinocytes. This layer is where keratinization of keratinocytes begins. Basal cells are undifferentiated, proliferating stem cells that give rise to keratinocytes, just above the basement membrane. This is the location of cell division. Daughter cells from the basal layer migrate upward and begin the process of differentiation; it takes 2 weeks to migrate from the basal cell layer to the top of the granular cell layer, and an additional 2 weeks for cells to cross the stratum corneum and shed.

Rete ridges are downward projections of the epidermis that help anchor the epidermis physically to the dermis.

The dermis is a tough, elastic support structure that contains blood vessels, nerves, and cutaneous appendages that provide structural integrity to the skin. Collagen and elastin fibers form the skeletal matrix, while extrafibrillar matrix forms the space between the fibers. The two dermal layers in descending order are the papillary dermis, in which collagen fibers are fine and loosely arranged, and the reticular dermis, in which collagen fibers are thick and densely packed; elastin fibers are primarily located here. Sensation is detected via free nerve endings and various sensory receptors depending on location. Free nerve endings are located in the epidermis of all skin and transmit information about pain and temperature. Meissner corpuscles are located in glabrous skin and are responsible for light touch and position sensation. Pacinian corpuscles lie within the deep skin layers, ligaments, and joints, and sense vibration and pressure. Merkel discs are present in the superficial skin of the fingertips and sense pressure, deep static touch, and position. Finally, Ruffini corpuscles lie in the fingertips and joints, and transmit information about pressure, slippage of objects along the surface of the skin, and joint angle changes.

Skin blood vessels supply nutrition and provide temperature regulation. Vasculature is generally arranged in two horizonal plexuses, the superficial plexus located at the lower border of the papillary dermis, and the deep plexus located in the reticular dermis. Shunts between the two plexuses are responsible for temperature regulation; increased blood flow in the superficial plexus allows heat loss, while increased flow in the deep plexus allows for heat conservation.

There are two types of sweat glands present in the integumentary system, eccrine and apocrine.

In total, 2–3 million eccrine sweat glands are distributed all over the body and are activated by both thermal and emotional stimuli, with secretion activated by the sympathetic nervous system (SNS). The eccrine gland is a coiled tubule in the dermis and empties into a sweat duct that ends in the epidermis. Sweat is initially isotonic but ends as a hypotonic solution due to reabsorption of electrolytes. Apocrine sweat glands are located in the axillary and anogenital areas and are responsible for body odor. The apocrine gland is a coiled tube in the dermis and drains its secretions onto the midportion of hair follicles.

Hair follicles are distributed over the entire body surface except the palms and the soles. The follicle is an invagination of the epidermis that contains a hair bulb, which is a population of rapidly dividing cells called the hair matrix that divide, differentiate, and form the hair shaft. Melanocytes in the matrix give the hair shaft its pigment.

Subcutaneous fat serves as insulation, absorbs trauma, and is an energy reserve. Aggregates of fat cells are separated by septa that contain blood vessels and nerves.

Fascia is made up of layers of connective tissues and serves to stabilize, impart strength, maintain vessel patency, separate muscles, and enclose organs [5]. There are two facial layers (superficial and deep), as well as visceral fascia that encloses organs, and parietal fascia that lines the walls of body cavities. Superficial Fascia is located directly under the superficial subcutaneous fat layer and is composed of loosely interwoven collagen and elastin fibers. Deep fascia is located around the bones, muscles, nerves, and blood vessels, is more fibrous than superficial, and contains hyaluronan. Deep fascia contains a rich vascular supply and nerve endings may be present. Beneath the deep fascia lies highly vascularized skeletal muscle which is innervated by its respective spinal nerves to provide functional movement and support.

## 3. Etiology of Pressure Injuries

Pressure injuries are caused by pressure, shear, or both. Pressure injuries result from prolonged immobility when soft tissues are compressed between bony prominences and external surfaces. Compression causes occlusion of the capillary bed; hypoxia leads to ischemia and subsequent necrosis [6,7]. Soft tissues between bony prominences and external surfaces are at risk of injury during prolonged immobility. In individuals with sensation and mobility intact, this pressure will trigger a feedback response that prompts the individual to change their position. In individuals lacking sensation and mobility, skin breakdown occurs. Body weight exerts a downward force on the tissue, inhibiting blood flow and resulting in tissue hypoxia; if pressure is sustained, tissue ischemia and necrosis occur [8]. Risk factors for pressure injury include prolonged immobility, sensory deficits, older age, poor nutrition, and excess moisture. Shear occurs when mechanical force works internally on the skin in a direction parallel to the skin surface [9]. Internal structures move in the opposite direction, leading to a deep tissue injury, and skin layers may separate and cause blood vessel tearing, leading to impaired blood flow. Risk factors for shear include immobility, repositioning an individual in bed, or a reclining seat. Elevation of the head of the bed higher than 30 degrees increases the likelihood of shear injury in the deep soft tissues present in the spine and hip juncture and may account for the number of sacral ulcers seen [10].

Notably, friction forces act parallel to the skin surface and damage the epidermis causing friction blisters but not pressure injuries. The resulting tissue injury is a superficial skin insult. Repetitive frictional forces on non-bony prominences can result in skin that is rough or hyperkeratotic with scaling, lichenification, and discoloration [10].

When an individual is repositioned to offload areas of ischemia, reperfusion injury may occur. Reperfusion causes increased reactive oxygen species in the tissue, which triggers inflammation. Continuous ischemic–reperfusion cycles are deemed more damaging than ischemic cycles alone [8].

The highest pressures occur at the interface between bone and muscle. Effects of tissue hypoxia are greatest at this depth as well, causing necrosis at this interface and leaving the skin intact. When an ulcer appears at the level of the skin, extensive deep tissue injury is likely to have already occurred [8].

## 4. Incidence, Prevalence and Cost

The incidence of pressure injuries is estimated to be approximately 1 to 3 million per year in the U.S. [8], and it is estimated that 20–50% of patients with new SCI will develop at least one hospital-acquired pressure injury during their acute hospitalization [11]. Further, the odds of acquiring the first pressure injury during the acute hospitalization for those with complete SCI appear to be 4.5 times greater than those with incomplete SCI [11]. During the first year after a SCI, the cumulative incidence of pressure injury is approximately 41 percent [12]. The prevalence of pressure injuries among all hospitalized patients is 5–15% overall, but is expected to be much higher in ICU patients. The 1999 National Pressure Ulcer Prevalence Survey found that the prevalence of pressure injuries was 15% overall and 25% in ICUs between 1999–2005 [8]. Patients with neurological disorders, particularly SCI, have a 25–85% lifetime risk of developing a pressure injury [11,13,14,15]. Darker-skinned patients have a higher risk of developing a pressure injury, possibly due to increased difficulty in identifying non-blanching erythema that occurs before skin breakdown [8].

Hospital acquired pressure injuries cost > 11 billion annually, with individual patient care cost ranging from USD 20,900 to USD 151,700 per pressure ulcer [10,16,17]. Medicare in 2007 estimated that each pressure ulcer adds USD 43,180 in costs to a hospital stay; as of 2008, the Centers for Medicare and Medicaid Services (CMS) have not paid for hospital-acquired pressure injuries in order to promote efficient pressure-injury prevention practices in hospitals [18].

## 5. Location and Severity of Pressure Injuries

Pressure injuries vary depending upon the setting and circumstances, but those acquired while lying in bed most commonly include sacrum, trochanters, heels, and feet, while those acquired while sitting usually include ischium heels and feet. Pressure injuries reported on admissions with SCI in Barcelona, Spain included 27% ischium, 21% sacrum, 20% trochanter, 6% heel, 6% foot, 4% malleolus and 2% perineal [19], whereas a more recent study of veterans with SCI from 16 centers in the U.S. demonstrated 48% ischial or perineal, 37% sacral, and 14.5% trochanteric injuries [20].

Less commonly, pressure injuries for those with long-term bed rest may occur at the olecranon process, occiput, shoulder, scapula, posterior scalp, and ears.

## 6. Risk Factors

Pressure injuries are most severe in at-risk populations, which includes any individual with impaired mobility and sensation due to SCI, neurological impairments, sedation, peri- or post-operative immobilization, hospitalization, and frailty. Other factors that increase severity include poor nutrition and natural skin aging [8]. Poor nutrition causes loss of muscle that normally acts as a barrier between bony prominences and external surfaces. Elderly individuals experience natural skin aging, which includes dermal and epidermal thinning, decreased epidermal turnover, and loss of dermal papillae that results in flattening of the dermoepidermal junction, increasing susceptibility to shear forces [8].

### 6.1. Risk Assessment Scales

Since the early 1960s, risk assessment scales have been in place across the globe. The Norton Scale, developed in 1962 in London, used physical condition, mental condition, activity, mobility, and incontinence scored from lowest function (1 = very bad) to highest function (4 = good) to identify patients at risk of pressure ulcers [18,21]. With a maximum score of 20 and lower scores indicating higher risk for pressure injuries, the authors established a recommended cutoff of 16, for which the sensitivity is 74.5% and the specificity is 70.6% [18,21].

Notably, a Modified Norton Scale [18,21] was developed in Sweden in 1987, with added variables including food and fluid intake, body temperature, and social activity providing a higher positive predictive (PPV) of 35.1% compared to 32.5% from the original Norton scale [18,21]. At a cutoff of 23, the sensitivity for the Modified Norton Scale is 77.8% and the specificity is 68.4% [18,21].

The Braden Scale using sensory perception, moisture, activity, mobility, nutrition, and friction/shear was developed in the 1980s in the U.S. to identify patients at risk of pressure injuries. As with the Norton and Modified Norton Scales, the first five variables listed are given a score from 1–4, with one indicating lowest functioning and four indicating highest functioning [18,21]. The “friction and shear” variable is given a score of 1–3, with one indicating lowest functioning and three indicating highest functioning. The maximum score is 23, and with a cutoff of 18, the sensitivity is 74.5% and the specificity is 73.7% [18,21]. A recent comparison between the Norton and Braden Scales demonstrated similar performance [22].

The first risk assessment scale for pressure ulcers specifically for persons with SCI was developed in 1996. The SCI Pressure Ulcer Scale (SCIPUS) analyzed 15 risk factors, including level of activity, mobility, presence of complete SCI, urine incontinence or moisture, autonomic dysreflexia/severe spasticity, age, tobacco use, pulmonary disease, cardiac disease/abnormal ECG, diabetes or glucose ≥ 110 mg/dL, renal disease, impaired cognitive function, living in a nursing home or hospital, albumin < 3.4 or total protein < 6.4, and hematocrit < 36.0% or hemoglobin < 12.0 g/dL [23]. Items are scored dichotomously (0 if absent, 1 or 2 if present) or have 3–5 options, with a maximum score of 25. Lower scores indicate better prognosis, such that a score of 0–2 is low risk, 3–5 is moderate risk, 6–8 is high risk, and 9–25 is very high risk [23]. A 2019 study published in *Nature* maintains that although SCIPUS maintains excellent sensitivity, specificity is poor and internal consistency is low. As a result, the authors recommend that the total score be used cautiously and that clinicians emphasize the qualitative aspects of the items rather than relying on scores [24].

Notably, the developers of the SCIPUS also created a new pressure ulcer risk assessment scale designed specifically for persons with paralysis who are living in a community setting with nine risk factors analyzed, including level of activity, level of mobility, presence of complete SCI, urinary incontinence or moisture, autonomic dysreflexia, pulmonary disease, renal disease, being prone to infections that cause breathing problems, and paralysis caused by trauma; this tool was called the SCI Pressure Ulcer Scale—Acute (SCIPUS-A) [25]. Similar to the SCIPUS, items are scored dichotomously (0 if absent, 1 or 2 if present) or have 3–5 options, with a maximum score of 25. Lower scores indicate better prognosis, such that 0–12 is low risk, 13–18 is moderate risk, 19–20 is high risk, and 21–25 is very high risk. 

### 6.2. Clinical Risk Factors

Two of the highest risk factors incorporated into risk assessment for pressure injuries include mobility impairments and sensory loss. The International Standards for Neurological Classification of Spinal Cord Injury (ISNCSCI) are the most validated and reliable measures for determining neurological impairment as medical history is obtained, as they provide the level and completeness of injury [26]. Higher levels and more complete SCI indicate less perception of sensation to alert the individual that tissue damage may be occurring, and fewer muscle groups to facilitate mobility and pressure relief [11,27,28]. Fewer active muscle groups also translate to markedly reduced muscle mass, and hence, protein reserve, that is needed to heal any damaged tissue [29,30,31]. A 10% loss of lean body mass leads to immune suppression and increases the risk of infection. A 15–20% loss of lean body mass delays wound healing [31]. Additionally, higher levels of injury are associated with neurogenic orthostatic hypotension (NOH) that reduces whole-body tissue perfusion, but also increases the risk of autonomic dysreflexia (AD) [32,33] and spasticity [34,35] that can provoke autonomic sweating and shear, respectively, both of which carry higher risk of pressure injuries. Thermoregulation is also markedly impaired after SCI. As such, the International Standards to document Autonomic Function following SCI (ISAFSCI) should be documented, particularly for individuals with SCI at or above T6 who are at high risk of AD [33]. Previous pressure injuries profoundly increase the risk of recurrence, with 36–50% persons with SCI who acquired a pressure injury developing recurrences [36,37]. It has been found that pressure injury history is a more viable measure of pressure injury outcome than measures taken at any single point in time over a brief period. Pressure injury recurrence in the person with a SCI has been associated with gender (male), age (older), race (African American), unemployment, residence in a nursing home, and previous pressure injury surgery [37,38].

### 6.3. Comorbidities

Pre-existing conditions are also associated with greater risk of pressure injuries and impair immune function, including advanced age, smoking, lung and cardiac disease, diabetes mellitus, renal disease, and impaired cognition [11,27]. Smoking and cardiovascular disease result in local and/or systemic ischemia and impair wound healing due to undersupplied oxygen and diminished levels of essential nutrients delivered to tissue. Patients with diabetes mellitus demonstrate deficient neutrophil chemotaxis, phagocytic, microbicidal activities, aberrant cellular infiltration and macrophage activation, decreased release of tumor necrosis factor (TNF-alpha), interleukin (IL-1beta), and vascular endothelial growth factor (VEGF) from macrophages, impaired leukocyte function, and decreased fibroblast proliferation [39].

Neurogenic obesity is present in 75–98% of persons with SCI [40,41], and in addition to adding weight that makes pressure relief more difficult, increased adipose tissue has been shown to markedly increase systemic inflammation by secreting proinflammatory cytokines, which increase the risk of tissue hypoxia and markedly impair tissue healing and repair [42]. Obesity increases the risk of impaired cutaneous wound healing, fascial dehiscence, surgical site infections, and vascular disease. Capillary density fails to meet the demand of adipose tissue because adipose tissue disproportionately outgrows capillary density. As a result, there is decreased tissue perfusion and oxygenation. In addition, metabolic demand is increased as the heart needs to sufficiently perfuse peripheral tissues in order to prevent ischemia [39].

### 6.4. Nutrition

Nutrition screening and assessment are necessary for identification of patients at risk of malnutrition and pressure-injury formation [29,43,44]. Neurogenic obesity in SCI is also associated with severe sarcopenia, especially in the lower extremities where most protein reserves are stored as muscle in persons who are neurologically intact [29,41,42]. While fats are an important energy reserve, carry fat-soluble vitamins, and provide insulation under the skin and padding of bony prominences, they promote systemic inflammation and impair wound healing, as discussed above. With markedly depleted protein stores, persons with SCI rapidly develop protein malnutrition with even minor wounds, even when provided with protein supplements and high-protein diets [43]. Traditionally, it is thought that pressure injuries require additional calories to compensate for the increased metabolic stress due to hypermetabolism. Malnutrition is defined as any nutritional imbalance, therefore even overweight or obese individual who experience a severe acute illness or traumatic event are at risk of malnutrition [45]. The National Pressure Ulcer Long-Term Care cohort study noted a higher pressure-injury incidence among frail residents, those with low body mass index, significant weight loss, and difficulty eating independently [46]. Weight loss and/or pre-existing malnutrition is a positive predictive variable for pressure-injury formation [47]. However, because metabolic rate in persons with SCI is 12–54% lower than those without SCI due to reduced lean mass, it is strongly recommended that indirect calorimetry be performed to guide caloric intake, as the usual caloric recommendation (30–35 calories/kg) for healing wounds likely does not apply to this population [29]. Overfeeding in SCI will result in worsening neurogenic obesity and metabolic syndrome, including diabetes mellitus, hypertension, dyslipidemia, and systemic inflammation [29,40,41,42]. As discussed above, lean body mass is essential for wound healing, as it represents a protein reservoir that contributes to all four phases of wound-healing, including hemostasis, inflammation, proliferation, and maturation. Protein is responsible for the repair and synthesis of enzymes involved in wound healing, cell multiplication, and collagen and connective tissue synthesis.

The amino acids glutamine, cysteine, and arginine are indispensable amino acids when the body is under stress [43]. Arginine stimulates insulin-like growth factor (IGF) that plays a role in wound healing and enhances collagen deposition. Protein, vitamin K, and vitamin C are essential for maintaining hemostasis and promote an inflammatory process [43]. Inflammation requires protein for cell proliferation and production of granulation tissue, while Vitamin A is necessary for synthesis of immune cells, and Vitamin C, an antioxidant, helps the wound transform from the inflammatory to the proliferative stage. Zinc is a cofactor for collagen formation, metabolizes protein, liberates vitamin A from storage in the liver, interacts with platelets in blood clotting, and assists in immune function [43]. Inadequate supplies of zinc can delay wound healing, but excess zinc can result in copper deficiency and subsequent reduction in lymphocyte synthesis. Copper is essential for preserving skin strength, blood vessels, and epithelial and connective tissue throughout the body [43]. In the proliferative protein is required to support collagen deposition, and the amino acids, arginine and glutamine, become conditionally essential. Vitamin A, a fat-soluble vitamin, facilitates the migration of macrophages in the wound, collagen synthesis and promotes epithelialization during this phase, while vitamin C, a water-soluble vitamin, contributes to collagen synthesis, iron absorption, activation of copper, and plays an important role in immune function and protein metabolism [43]. Fat-soluble Vitamin D is involved in cellular differentiation and proliferation. Iron and protein are required for hemoglobin synthesis. Carbohydrates/glucose support cell growth, fibroblasts, and leukocytes, and serve as the main source of fuel for collagen synthesis. During the maturation phase, adequate energy, protein, and micronutrients continue to be essential, as outlined above [29,43,44].

It is estimated that 1.25–1.5 g/kg of daily protein intake is required to facilitate pressure-injury healing. A 12-week RCT found that pressure-injury healing accelerated when a nutritional formula enriched with protein, arginine, zinc, and antioxidants was consumed daily for 8 weeks compared to an isocaloric–isonitrogenous formula. Increased healing rates were associated with the arginine-enriched formula that appeared to be cost-effective, reducing direct nursing care, treatments, and dressings [43].

Anemia is a condition in which hemoglobin concentration and/or red blood cell numbers are lower than normal and are insufficient to meet an individual’s physiological needs [44]. As hemoglobin is a protein entity dependent upon adequate protein stores within the body, it can be depleted in conditions of protein malnutrition, as described above. When anemia is present, the blood has reduced oxygen-carrying capacity, causing decreased immune function and tissue hypoxia and further impairing wound healing [45]. One study of long-term-care patients has shown that anemia is associated with non-healing pressure injuries over 6 months [46]. Another study found that low hemoglobin on admission is associated with higher incidence of pressure injuries after hip fracture [47].

Excessive moisture may lead to maceration of the skin. Sources of moisture that cause a predisposition to skin breakdown include perspiration, urine, feces, and fistula or wound drainage. Increases in pressure-injury risk related to moisture are due to an increase in friction that occurs between the skin and textiles or due to an increase in bacterial load that occurs because alkaline sources of moisture neutralize the protection provided by the normal acidity of the skin [48].

## 7. Wound Assessment

**Figure 1:**
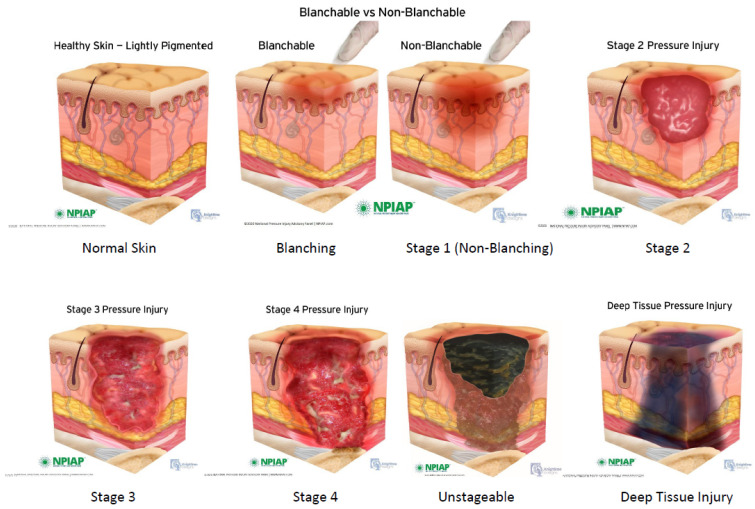
Normal skin and pressure injuries of Stages 1–4, Unstageable and Deep Tissue Injury. Used with permission from the National Pressure Injury Advisory Panel (NPIAP). Copyright 2022 NPIAP.

The Pressure Injury Staging System has recently been revised and updated by the National Pressure Ulcer Advisory Panel (NPUAP) [49] (Figure 1). Non-blanchable erythema of intact skin, i.e., intact epidermal layer, described as a *Stage 1* pressure injury, may be challenging to detect in darkly pigmented skin. A *Stage 2* pressure injury is characterized by partial-thickness skin loss with exposed dermis, and may present as an abrasion, blister, or shallow crater. The wound bed is pink/red and moist. Adipose and deeper tissues are not visible, and granulation tissue, slough, or eschar are not present. A *Stage 3* pressure injury involves full-thickness skin loss, with visible adipose tissue; granulation tissue is often present. Fascia, muscle, tendon, ligament, cartilage, or bone is not exposed. If heavy slough or eschar are present, the wound is considered unstageable. *Stage 4* pressure injuries have full-thickness skin and tissue loss involving fascia, muscle, tendon, ligament, cartilage, or bone. Slough or eschar may be visible. Rolled edges, undermining, and tunneling often occur. If slough or eschar obscure the extent of tissue loss, the wound is considered *Unstageable*. If slough or eschar is removed, a Stage 3 or 4 pressure injury will be revealed. A *Deep Tissue Injury* is described as persistent non-blanchable deep red, maroon, or purple discoloration. It may evolve rapidly to reveal the actual extent of tissue injury or may resolve without tissue loss.

## 8. Wound Bed Assessment

When describing a pressure injury, the location and size of the wound should be noted, including length, width, and depth; ideally, a picture grid with accurate measurements would be provided. The type of tissue at the base of the wound provides useful information about wound-healing time and potential complications. Healthy granulation tissue is bright beefy red in color and indicates healing skin and the growth of new blood vessels. Unhealthy granulation tissue is dark red or pale pink in color, bleeds on contact, and may indicate infection. Necrotic tissue, slough, and eschar impede healing [50]. Specific terms for communicating about wounds have been reviewed and provided in a glossary on behalf of the NPUAP; briefly, those terms are provided here [28]. Biofilm is a collection of complex microbial communities containing bacteria and fungi attached to a living or non-living surface by a protective matrix. An eschar is a collection of dead tissue appearing black or brown, dry, thick, and leathery. Slough is a light yellow/cream-colored, moist, soft inflammatory exudate composed of proteinaceous tissue, fibrin, neutrophils, and bacteria. Additional terms that should not be used with pressure injuries include incontinence-associated dermatitis (IAD), medical-adhesive-related skin injuries (MARSI), and moisture-associated skin damage (MASD) [50].

## 9. Wound Assessment Tools

Using a Triangle of Wound Assessment, the wound is divided into three components: the wound bed, wound edge, and the peri-wound skin, with each described fully as above [51]. The Bates-Jensen Wound Assessment Tool rates 13 aspects of the wound, including size, depth, edges, undermining, necrotic tissue amount, exudate type, exudate amount, skin color surrounding wound, peripheral tissue edema, peripheral tissue induration, granulation tissue, and epithelialization [52]. The higher the total score, the more severe the wound status. The TIME acronym (Tissue management, Infection control, Moisture imbalance, Edge of wound) was developed in 2002 by a group of wound care experts promoting four clinical observations to be considered and paired with appropriate wound bed preparation clinical actions. The specific clinical observations include non-viable or deficient tissue, infection or inflammation, moisture imbalance, and edge of wound non-advancing or undermining [52,53].

## 10. Prevention

A 2020 study of Australian hospital networks depicted that a great proportion of hospitals did not have any publicly available patient education materials on PI prevention. Sites that did have materials contained accurate information on PI risk factors and defining characteristics, but information outside these was deficient. They concluded that there is a significant deficit in the availability of education materials for acute care patients and their families [54]. Freely available resources are needed for patient engagement in their wound-healing process, as the recent systematic review reported that complex intervention programs that included patient education were more effective at decreasing the occurrence of hospital-acquired pressure injuries than single interventions [54].

### 10.1. Acute Care Support Surface

As discussed above, education of staff and patients should include a multidisciplinary approach and consider all aspects of care that may prevent pressure injuries beginning with the support surface. A support surface is a specialized device of pressure redistribution designed for the management of tissue loads, microclimate, and other therapeutic functions. It functions to redistribute the body’s weight and protect the skin’s tissue while providing for proper body alignment, comfort, and as part of a seating system, postural control during functional movement. Support surfaces include mattresses, integrated bed systems, mattress replacements, mattress overlays, and seat cushions. Achieving a proper match between the patient’s needs and the performance capabilities of the support surface has a profound positive impact on a patient’s health and well-being. The Wound Ostomy Continence Nursing (WONC) Society recently created an evidence-based support surface algorithm [55]. Preventive support surfaces are recommended for those at risk, i.e., Braden Score ≤ 18 (Table 1).

### 10.2. Repositioning

Proper positioning is accomplished through properly chosen support surfaces, adequate periodic pressure redistribution, and protection of bony prominences such as the heels, sacrum, and coccyx [56].

EPUPAP/NPIAP/PPPIA guidelines for positioning and repositioning individuals in bed include: (1) Reposition to relieve or redistribute pressure using manual handling techniques and equipment that do not produce excessive shear strain and shear force; (2) use the 30 degree side-lying position in preference to the 90 degree side-lying position; (3) encourage individuals who can reposition themselves to sleep in a 20–30 degrees side-lying position or flat in bed if not contraindicated; (4) avoid extended use of prone positioning unless required for management of a medical conditioning; and (5) keep the head of bed as flat as possible [1]. Despite the common perception that critically ill patients are “too unstable” to turn or reposition, small shifts in position can be accomplished with careful, incremental movement that allow offloading of pressure from skin and bony prominences. 

### 10.3. Wheelchair Seating Systems

Risk of pressure injury is higher in wheelchair users than the general population due to protracted periods of time spent seated without pressure relief. In the seated individual, body weight is loaded onto a relatively small surface area, namely the ischial tuberosities and buttocks, the coccyx, and the upper thighs. Hence, pressure injuries occur most commonly around the bony prominences in these weight-bearing areas [57]. Pressure injuries that develop in the seated individual cause significant distress because treatment involves offloading the affected area in order to promote tissue perfusion through bed rest. Bed rest has been found to have negative psychosocial effects such as depression. Choosing the correct wheelchair seating system is essential for reducing the risk of pressure-injury formation. No cushion can perform effectively in the prevention of pressure injuries if the wheelchair is not properly fitted, including: (1) chair width, such that the seat is as small as possible, with just a finger width space on each side between the body and side supports. If the chair is too narrow, a pelvic obliquity may occur which rotates the spin and creates seating instability, also increasing the weight bearing on one of the buttocks which can lead to increased PI risk. (2) The chair depth should be such that when the buttocks are in contact with the backrest, there should be just a few fingers’ space between the front of the seat and the individual’s calf. If the seat is too long, the contact between the person’s calves and the seat will make the user slide forward in the seat, causing increased shear forces in the buttocks and putting pressure on the coccyx. If the seat is too short, the area over which the pressure is distributed is reduced, increasing the pressure on vulnerable areas. (3) The chair height between the top of the seat cushion and the footplate or floor should be identical to the distance of the heel to the back of the knee. If the feet are not supported, the person will lose stability and slide down in the chair, creating a slouched position and increased pressure on the coccyx. The position of the feet should allow the knees to be at 90 degrees. (4) The backrest should be designed to follow the shape of the back in order to increase the contact area and reduce pressure on the seated area while also reducing the risk of sliding forces. (5) The tilt mechanism allows the seat to floor angle to be actively and frequently changed and is advised for most wheelchair users with reduced upper extremity mobility. American chairs have a seat tilt from 0° to 40–60°, enhancing offloading of the pressure areas. (6) Cushion selection and evaluation should be performed by a seating specialist [58]. The seating evaluation should include mat and functional assessment. The mat examination helps to determine the functional postural limitations of the spine, pelvis, and extremities, and to determine appropriate measurements for wheelchair fitting. The functional examination is to determine the seating and mobility needs of the individual in the immediate, intermediate, and community environments. Pressure-redistributing wheelchair cushions designed to maintain tissue integrity reduce the incidence of sitting-induced pressure ulcers and are more effective for preventing ulcer injury than the standard segmented foam cushions [59]. Patients in wheelchairs also require shifts in body weight. The recommendation is that the patient is taught to shift their weight 30 s every 15 min and reposition every hour [1].

### 10.4. Assistive Technology/Adaptive Equipment

Assistive devices are external devices that are designed, made, or adapted to assist a person to perform a particular task. Many individuals with disabilities depend on assistive devices to enable them to carry out daily activities and participate actively and productively in their communities. Examples include mobility devices (wheelchairs, tricycles, crutches, walking canes, walkers), positioning devices (wedges, cushions, standing frames), prosthetic devices, and daily living devices (adapted cutlery and cups, shower seats, toilet seats and frames, commodes, dressing sticks) [60]. Four assistive technologies that have been found to support self-management of PIs in SCI patients include: (1) computer-based educational technologies that improved short-term gains in knowledge; (2) interface pressure mapping technologies that improved adherence to pressure-relief schedules up to 3 months; (3) electrical stimulation of at-risk areas, which improved tissue tolerance after 8 weeks of training; and (4) telemedicine programs that improved independence and reduced hospital visits over 6 months [61].

## 11. Wound Healing

### 11.1. Hemostasis

Collagen plays a critical role in all stages of wound healing [62,63]. When injury occurs to the skin, collagen is exposed and activates the clotting cascade. Platelets are recruited and degranulate, activating factor XII (Hageman factor) and releasing cytokines such as platelet-derived growth factor (PDGF), transforming growth factor (TGF), and epidermal growth factor (EGF). These cytokines, along with tissue debris (such as collagen fragments), attract neutrophils. A fibrin clot is formed. This clot serves as a preliminary matrix in the wound space that enables cells to migrate into the region. In time, fibrinolysis occurs and allows ease of further cell migration into the wound space; the duration of this phase lasts seconds to hours [64].

### 11.2. Inflammation

Collagen I and IV debris recruit inflammatory mediators (neutrophils and macrophages) that phagocytose debris, and fibroblasts that synthesize collagen and the extracellular matrix (ECM). The inflammatory stage of wound healing is dependent on innate immune-system activity that involves macrophages, neutrophils, interferons, interleukins, mast cells, acute-phase proteins, as well as the physical barrier provided by the stratum corneum [65]. Pathogen associated molecular patterns (PAMPs) and damage-associated molecular patterns (DAMPs) are recognized by Toll-like receptors and the innate immune response is rapidly activated [65]. Neutrophils appear shortly after injury and reach their peak number within 24–48 h. Macrophages arrive at the site of injury within 2–3 days, and in addition to their phagocytic ability, they release cytokines such as TNF-alpha and interleukins that are essential for wound healing; lymphocytes follow macrophages to the site of injury. Damaged ECM proteins consisting of collagen must be removed to accommodate newly synthesized collagen that is properly aligned in the matrix and allows migration of epidermal cells and fibroblasts. Collagenases are responsible for the degradation of collagen into small fragments that are then removed by neutrophils and macrophages [62,63]. The C-propeptide fragment of collagen I attracts endothelial cells, triggering angiogenesis. Additionally, keratinocytes are recruited to the area and contribute to wound re-epithelialization; the duration of this phase is hours to days [64].

### 11.3. Proliferation

Proliferation begins 3 days after injury and is characterized by fibroblast proliferation and growth factor synthesis that enables the formation of granulation tissue. Collagen degradation during injury promotes fibroblast proliferation [63,64]. Fibroblasts reach peak levels around day 7 after injury and synthesize and deposit extracellular proteins that form the ECM. The ECM is made up of fibrous proteins, including collagen, elastin, and adhesive proteins such as laminin and fibronectin, and polysaccharides such as proteoglycans and glycosaminoglycans (GAGs). Granulation tissue is made of collagen III. Vascular endothelial cells arise from the damaged end of vessels and capillaries and play a key role in angiogenesis. New vessels originate as capillaries that sprout from damaged vessels at the wound edge. Matrix metalloproteinases (MMPs) assist in angiogenesis by degrading the basement membrane that surrounds vascular endothelial cells, allowing capillary buds to sprout and erode the ECM. Epithelialization occurs when marginal basal cells alter their adhesion properties and migrate from the underlying epidermal–dermal junction to the provisional matrix. The proliferation phase closes with wound contraction. The contractile ability of fibroblasts and myofibroblasts shrinks granulation tissue and pulls on wound edges, leading to contraction of the wound [62,63]. The duration of this phase is days to weeks [65].

### 11.4. Maturation

The maturation phase is characterized by tissue remodeling and development of tensile strength, and typically starts 7 days after injury occurs. The provisional ECM is predominately made of fibronectin and serves two purposes: to act as a substratum for cell migration and subsequent growth and as a template for collagen deposition. Collagen then serves as the predominant constituent of the matrix and forms fibrillar bundles that provide stiffness and tensile strength to the wound [62,63]. This phase closes with scar formation, which is an acellular and avascular mass of collagen that serves to restore tissue continuity. The duration of this phase is days to weeks months/years [64].

## 12. Chronic Non-Healing Wounds

Chronic wounds are the result of a halt in the inflammatory phase of wound healing. There are two potential mechanisms: (1) signals such as growth factor production or keratinocyte proliferation are not generated; and/or (2) the inflammatory phase is not able to be completed, and as a result, bacterial contamination is not cleared [64,66]. A prolonged inflammatory phase is due to neutrophils and their production of the proinflammatory cytokines that perpetuate inflammation. In addition, release of tissue-damaging proteinases such as MMPs can degrade newly formed tissue and delay the wound-healing process. Chronic wounds are characterized by high numbers of Langerhans cells, neutrophils, pro-inflammatory macrophages, and proteases linked to clinical severity [64]. Loss of appendages in full-thickness wounds cause impaired re-epithelialization. Normally, epithelial cells use the smooth, moist surface of the basement membrane to move across the wound. In chronic wounds, epithelial cells latch onto and pull themselves across the scaffolding of macromolecules of the provisional matrix.

## 13. Local Factors Impair Healing

A number of local factors can impair wound healing, including hypoxia, infection, and inflammation [67]. Oxygen is critical for all wound-healing phases. It helps prevent wound infection; induces angiogenesis; increases keratinocyte differentiation, migration, and re-epithelialization; enhances fibroblast proliferation and collagen synthesis; and promotes wound contraction. The microenvironment of the early wound is hypoxic due to the vascular disruption and high oxygen consumption of metabolically active cells [64,66]. Systemic conditions such as aging and diabetes create impaired vascular flow and contribute to hypoxia. In healing wounds, hypoxia induces cytokine and growth factor production from macrophages, keratinocytes, and fibroblasts. The reactive oxygen species (ROS) act as cellular messengers to stimulate wound-healing processes, including cell motility, cytokine action, and angiogenesis. Hence, hypoxia is needed to stimulate wound healing, but oxygen is needed to sustain the healing process. Infections occur when injured skin enables access to underlying tissues. Inflammation is a normal part of the wound-healing process and is critical in removing microorganisms. When decontamination is inefficient, the inflammatory phase is prolonged, as microorganisms have yet to be cleared. Bacteria and endotoxins lead to prolonged elevation of pro-inflammatory cytokines such as IL-1 and TNF-α that prolong the inflammatory phase. This leads to increased MMPs, which increases the protease content and degrades growth factors. Pseudomonas aeruginosa and Staphylococcus aureus create a biofilm in a wound that shields bacteria from PMNs.

## 14. Systemic Factors Impair Healing

With advancing age, delayed wound healing is associated with an altered inflammatory response, such as delayed T-cell infiltration into the site of injury with alterations in chemokine production and reduced macrophage phagocytic activity. Other features include delayed re-epithelization, delayed angiogenesis and collagen deposition, reduced collagen turnover and modeling, and decreased wound strength [39,64,67]. Sex hormones also play a role, as compared to aged females, aged males have delayed healing. Studies indicate that estrogen can improve the age-related impairment in healing in both men and women, while androgens regulate cutaneous wound healing negatively. Stress and certain medications including glucocorticoids, non-steroidal anti-inflammatory drugs (NSAIDs), and chemotherapeutic drugs can impair wound healing. Glucocorticoids inhibit wound repair via global anti-inflammatory effects and suppression of cellular wound responses, including fibroblast proliferation and collagen synthesis. Systemic steroids cause wounds to heal with impaired granulation tissue and reduced wound contraction.

It appears that NSAIDs have an anti-proliferative effect on wound healing, resulting in decreased numbers of fibroblasts, weakened breaking strength, reduced wound contraction, delayed re-epithelization, and impaired angiogenesis. Finally, many chemotherapeutics inhibit cellular metabolism, rapid cell division, and angiogenesis, thus affecting may pathways critical in the wound-healing process [39,64,67]. Diabetes is associated with hypoxia that impairs wound healing and angiogenesis through decreased levels of VEG-F, as well as hyperglycemia, which adds to oxidative stress. Delayed bacterial clearance also occurs. As mentioned above, obesity impairs wound healing through secretion of proinflammatory cytokines, including TNF-α, IL-1, Il-6 and nuclear factor kappa-light-chain-enhancer of activated B cells (NFκB), as well creating hypoperfusion and ischemia in subcutaneous tissue. Alcohol consumption increases host susceptibility to wound infection, and smoking causes impaired white blood cell migration into the site of injury during the inflammatory phase, resulting in lower numbers of monocytes and macrophages in the wound site and reduced neutrophil bactericidal activity [39,64,67].

## 15. Pressure-Injury Treatment

**Figure 2:**
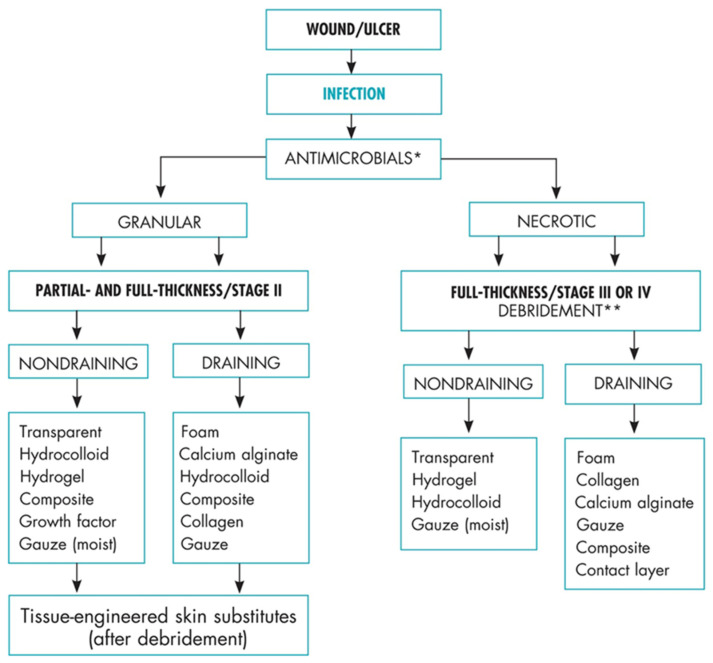
Wound treatment algorithm. * Antimicrobials may be topical or within a dressing component. ** Debridement may be done by surgical, mechanical, autolytic, or enzymatic methods. Adopted with permission from (©Baranoski, S., McIntosh, A., Montoya, L. Optimizing the use of Dressing, Lecture, Clinical Symposium on Advances in Skin and Wound Care. Las Vegas, NV, September 2014.).

First and foremost, pressure-injury healing requires pressure relief, so that oxygen and nutrients can be supplied to the site, and the underlying cause of the pressure injury must be determined and corrected. The wound bed must be clean, without eschar, slough, or biofilm, so that new epithelial tissue can emerge. Underlying osteomyelitis/infection must be eradicated, nutrients must be made available, and a good vascular supply is essential. An interprofessional approach to the management of wounds has been shown to increase healing and decrease recurrence [68]. Members of the interdisciplinary team include registered nurses, registered dietician nutritionists, physical therapists, occupational therapists, wheelchair seating specialists, social workers, mental health specialists, and physicians. Assessments should include nutritional screening, risk of pressure-injury development (Norton, Braden Score, SCIPUS), support surface/positioning assessment, seating assessment (i.e., mat examination and functional assessment), wound assessment (e.g., Triangle of Wound Assessment, Bates-Jensen, and TIME), pain assessment, mental health screening (e.g., CWQoL, PHQ9, GAD7), access to resources (Social Determinants of Health screening tool) [69] and debridement/biofilm reduction and cleansing [70]. A wound treatment algorithm may guide treatment strategies (Figure 2) [71].

Nutrition counseling is essential to optimize the intake of nutrient dense foods and supplements with low caloric density in order not to contribute to exacerbating neurogenic obesity [29,42]. A high-potency multivitamin and mineral supplement should be provided daily [72]. Ideally, basal energy expenditure should be assessed by indirect calorimetry and total daily energy expenditure determined to ensure overfeeding does not occur [73]. Protein-dense supplements with low caloric density, such as arginine-based products, should be provided to meet the 1.5 g/kg body weight recommendations of EPUAP/NPIAP/PPPIA Clinical Practice Guidelines [1]. Of note, prealbumin (also known as transthyretin), a biomarker of recent (3–5 days) protein nutrition, fell into disfavor in 2012 when the Academy of Nutrition and Dietetics and the American Society for Parenteral and Enteral Nutrition discouraged its use as a “sole” indicator for undernutrition due to its susceptibility to systemic inflammation [74]. Recently, however, the Global Leadership Initiative on Malnutrition (GLIM) has endorsed prealbumin as an important indicator of acute protein status so long as C-reactive protein (CRP) is <15 mg/dL; if CRP exceeds this number, prealbumin levels are uninterpretable [75,76,77].

The Wound Bed Preparation (WBP) paradigm is a structured approach to wound healing that focuses on optimizing conditions at the wound bed to encourage normal, endogenous healing [78]. Debridement is defined as the removal of necrotic tissue, exudate, bacteria, and metabolic waste from a wound in order to improve or facilitate the healing process. Dead and necrotic tissue needs to be removed because it is a pro-inflammatory stimulus and a culture medium for bacterial growth (biofilms). Debridement creates an acute wound within a chronic wound, restoring nutrient supply from the underlying circulation and facilitating optimal available oxygen delivery to the wound site [70]. Cleansing should be performed with normal saline solution or low-cytotoxicity cleansers [79].

## 16. Wound Infections/Osteomyelitis

When necrotic tissue is not removed, the spread of bacterial damage to deeper tissue may occur, causing surrounding cellulitis, osteomyelitis, and the possibility of septicemia, preventable limb amputation, or death [70]. The wound bioburden cycle includes *contamination,* in which organisms are present on the wound surface without no signs or symptoms; *colonization,* in which organisms multiply on wound surface with early reversible adherence, then later, irreversible adherence of organisms to the tissue without signs or symptoms; *critical colonization/biofilm,* in which organisms form biofilm on the wound surface, creating signs and symptoms of delayed wound healing and local infection, followed by frank *infection,* in which organisms invade tissue and systemic response occurs, with signs and symptoms of fever, leukocytosis, and inflammation (i.e., erythema, warmth, increased exudate, pain, and fever). Sibbald and colleagues proposed assessment for critical colonization diagnostic criteria using the acronym NERDS (non-healing wound, exudative wound, red and bleeding wound, debris in the wound, and smell from the wound) or STONES (size is bigger, temperature is increased, osteomyelitis, new areas of breakdown, exudate, erythema, or edema, and smell) for infection, respectively [78,80]. In addition, signs and symptoms of infection include purulent drainage, pain, tenderness, localized swelling, redness, and heat, but also increased spasticity [34,35] and AD for those with SCI above T6 [32].

To manage bioburden colonization in the absence of infection, an ideal antiseptic has low toxicity and is effective at killing microbes but does not impact wound healing. Common antiseptics used to control bioburden and biofilm include acetic acid, chlorhexidine, Ethylenediaminetetraacetic acid (EDTA), honey, iodine, lactoferrin, methylene blue-gentian violet, mupirocin, polyhexamethylene biguanide, potassium permanganate, silver, and xytol.

Data comparing these antiseptics are lacking, such that clinical judgement and local preference should be exercised [66].

The gold standard for diagnosing osteomyelitis is microbiological identification by bone biopsy, but plain X-rays, white cell count, and erythrocyte sedimentation rate are 89% sensitive and 88% specific for diagnosing osteomyelitis in a non-invasive workup [81]. Approximately 25% of non-healing wounds are associated with osteomyelitis and will not heal until the bacteria has been eradicated. Nonetheless, empiric antibiotic treatment without deep wound or bone biopsy is discouraged. Biofilm can adapt to antibiotics by reducing their metabolism, oxygen consumption, and nutrient requirements. Empiric antibiotic treatment can be used initially but molecular diagnostic techniques used to select systemic antibiotics are strongly recommended to maximize the effect of offending organisms and mitigate the risk of antibiotic resistance [67,82,83].

Once diagnosed, debridement of the infected bone and 6 weeks of appropriate antibiotics are essential to completely treat osteomyelitis [84,85].

## 17. Dressings

Dr. George Winter, cited as the father of moist wound healing, completed a landmark study in the 1960s, comparing the effect of air drying versus occlusive dressings on epithelialization in an animal model [86]. Due to his work, it is known that wound healing requires a moist environment that enables epithelial cells to migrate from wound edges to re-epithelialize the wound. Ideal dressings provide moisture balance. Characteristics of an ideal dressing include dressings that maintain a moist environment, facilitate autolytic debridement, are absorbent, provide thermal insulation, act as a bacterial barrier, and have pain-free removal [71]. The NICE acronym (Necrosis, Infection/Inflammation, Characteristics, Exudate) can be used to determine the most appropriate dressing to use based on the wound presentation. For necrotic tissue, slough, or eschar, wet-to-dry dressings are a non-selective method of mechanical debridement. Autolytic debridement of tissue is best accomplished with hydrogels, hydrocolloids, and alginate dressings.

With dressing-stimulated autolytic debridement, it is essential to monitor for secondary infection and remove unwanted slough with each dressing change. If infection/inflammation is present, consider using antimicrobial dressings such as silver or iodine; infected wounds may require more frequent dressing changes. A dressing should be selected and reassessed based on location and characteristics of the wound, such as the use of conformable dressings for hard-to-fit areas.

Waterproof dressings may be used if incontinence is an issue. The patient’s pain (and or AD and spasticity) should be considered, and dressings that may promote comfort and pain reduction should be selected. If an exudate is present, the absorbency of the dressing (non, low, moderate, heavy) should be matched to the amount of exudate from the wound. The surrounding skin should be continually assessed for macerations.

Primary dressings are those that come into contact with the wound bed and fall within three categories: (1) those that maintain adequate moisture, (2) those that absorb excess moisture, and (3) those that add moisture. Secondary dressings are those that cover a primary dressing or secure a dressing in place. There are multiple dressing types to consider, including transparent film, hydrocolloid, hydrogel, honey, foam, calcium alginate, composite, collagen, contact layer, antimicrobial, and absorptive dressings. *Transparent film* dressings are indicated for superficial wounds with absent or low levels of exudate and imitate the outer skin layer to provide a moist environment, allowing epithelial cells to migrate over the surface of the wound. Fluid may accumulate under the dressing, which is a useful adjunct to create an autolytic environment, thereby inducing a cleaner wound surface. If excess fluid accumulates or leaks out from the sides, the dressing needs to be changed, as maceration can occur if the dressing is not changed in a timely manner. Most films can be left in place for up to 7 days. These dressings can also be used for healthy skin (not aging or fragile skin) and serve as excellent secondary dressings.

*Hydrocolloid dressings* are composed of opaque mixtures of adhesive, absorbent polymers, pectin gelling agents, and sodium carboxymethylcellulose. They are impermeable to gases and water vapor and form a soft gel over the wound bed, such that odor during dressing changes is normal. These dressings provide a moist environment that is conducive to autolytic debridement. They are often used as a preventive dressing on high-risk areas such as the sacrum and heels to protect skin from frequent tape removal. They are indicated for minimal to moderately heavy exudating pressure injuries. Notably, hyper-granulation tissue and maceration may occur if dressing changes do not occur in a timely manner (typically 3 to 7 days depending on the manufacturer. *Hydrogel dressings* are efficient at hydrating or donating moisture to dry wound beds, as well as softening and loosening slough and necrotic wound debris. Hydrogels are available as amorphous gels, 3D sheets, or amorphous gels impregnated into other mesh-type dressings. Maceration can be a concern, so the periwound area needs to be protected from excessive hydration; protective barriers are often recommended. *Honey (active Leptospermum)* dressings have multiple mechanisms of action that include reducing edema, lowering wound pH, and promoting autolytic debridement. Honey-containing products are available in gels, sheets, contact layers, and as a hydrocolloid, and are indicated for infected or highly colonized wounds and wounds with slough or necrosis. They may also be used for partial and full-thickness wounds. *Foam* dressings are highly absorbent dressings made from a polyurethane base and are permeable to both gases and water vapor. Their hydrophilic properties allow for absorption of exudate into the layers of the foam. Foam dressings are indicated for wounds with moderate to heavy exudate, and for prophylactic protection over bony prominences or friction areas. They can be used as a primary or secondary dressing, can be left on for 3 to 5 days, and are effective for granular wounds, skin tears, donor sites, under compression wraps, and hypergranulation tissue. *Calcium alginate* dressings are absorbent, non-adherent, biodegradable non-woven fibers derived from brown seaweed, and are composed of calcium salts of alginic, mannuronic, and guluronic acids. They are useful for highly exudative wounds. When alginate dressings come into contact with sodium-rich solutions such as wound drainage, the calcium ions undergo an exchange for sodium ions, forming a soluble alginate gel that maintains a moist wound bed and supports a therapeutic healing environment. They can absorb 20 times their weight and also have hemostatic properties. They are usually changed daily or as indicated by the amount of drainage. One disadvantage is that these dressings can dry out and adhere to the wound bed but using an appropriate secondary dressing that helps to maintain a gelling state can combat this. *Composite* dressings are made up of a combination of materials and serves as a bacterial barrier or absorptive layer. Not all composite dressings provide a moist environment, and many require a secondary dressing to create a moist environment, referred to as an “island dressing.” *Collagen* dressings should be considered when the wound fails to progress through the normal wound-healing process. Delayed wound healing is commonly the result of an extended inflammatory phase often caused by MMPs in the wound that were initially necessary to break down damaged tissue, but when too many are present, they destroy healthy ECM and impede wound healing. Collagen dressings can restart the wound-healing cascade, support a moist wound-healing environment, encourage the deposition of new collagen fibers, and support new tissue growth and granulation tissue growth. These dressings are derived from bovine, ovine, porcine, equine, or avian tissues and can be 100% collagen or combined with alginates or other products. Collagen products can be in sheet form or gels, are typically changed every 1 to 3 days, and require a secondary dressing for securement. *Contact-layer* dressings are a single layer of a woven net that acts as a low-adherence material when applied to wound surfaces. A contact layer dressing is applied directly to the wound and acts as a protective interface between the wound and the secondary dressings. Their main purpose is to allow exudate to pass through the contact layer and into the secondary dressing. The secondary dressing is changed more frequently, but the contact-layer dressing can be left in place for up to 7 days. *Antimicrobial* dressings provide the benefit of an activated antimicrobial effect against bacteria and a moist environment for healing. The active ingredient may be silver ions, cadexomer iodine, honey, or polyhexamethylene biguanide, but they do not replace the need for systemic antibiotic therapy.

Antimicrobial dressings are available in a variety of forms, including transparent dressings, alginates, contact layers, gauze, island dressings, foams, and absorptive fillers. Silver dressings are indicated to reduce bioburden in colonized or infected wounds. *Absorptive* dressings are multilayered and include a semi-adherent or a non-adherent layer combined with highly absorptive layers of fibers such as cellulose, cotton, or rayon that effectively manage exudate. They are best used for moderate to heavy draining wounds and can be either a primary or secondary dressing. Their main function is to minimize adherence to the wound bed and manage the exudate.

## 18. Negative Pressure/Wound Vacuum

Negative Pressure Wound Therapy (NPWT) is used to assist in wound closure, as the externally applied negative pressure creates macrostrain (the physical response seen as the negative pressure contracts the wound) and microstrain in the wound bed and in individual cells [87]. Microstrain is a mechanical stress that creates changes at the cells’ surface called microdeformations that cause growth factors and cytokines to upregulate fibroblastic activity, increasing the production of the ECM and cell proliferation within the wound and ultimately creating new granulation tissue [88]. The NPIAP/EPUAP/PPPIA guidelines recommend the use of this modality for the treatment of deep, stage 3 and stage 4 PIs [1]. NPWT applies suction to the wound bed through the use of a device that is attached to a wound contact layer (interface dressing) through a plastic tube. Most clinicians use a foam interface dressing and interface dressings are covered with a transparent film that seals the wound and dressing to maintain the vacuum effect. It is recommended to initially change the dressing 48 h after beginning treatment, and then 2 to 3 times per week.

NPWT facilitates wound closure and healing through several mechanisms of action, including facilitating wound contraction/retraction; removing edema, and thereby improving nutrient and oxygen delivery; removing exudate, which may be a medium for bacterial colonization; decreasing harmful levels of proinflammatory agents such as MMPs found in chronic wounds; and promoting angiogenesis [1].

## 19. Electrical Stimulation

The utility of electrical stimulation (ES) in wound healing was discovered in the 18th and 19th centuries when Galvani and Moruzzi made cut preparations of sciatic nerve and muscle tissue and found that small electrical currents were generated at the cut points, now referred to as injury potentials [89]. It was later found that these currents disappeared when wounds healed, showing that ES can “jump-start” the stalled wound healing. ES can be used for stage 3 or 4 PIs and is considered one of the most cost effective and efficacious tissue-repair and wound-healing accelerators [1]. Unfortunately, it is not widely used due to non-standardized clinical algorithms and lack of knowledge, education, and training in the application of this energy. A recent systematic review and meta-analysis of 599 articles yielded 15 that met standardized inclusion criteria, and only 4 of those included randomized controlled trials (RCTs) of ES on the number of healed events [90]. Among those RCTs, healing of a pressure injury with ES was found to be 1.55 times more likely than standard wound care or sham ES [90]. The benefits of ES include increased blood flow, increased tissue oxygenation, increased fibroblast proliferation and collagen deposition, increased angiogenesis, decreased wound pain, and increased wound tensile strength [71]. High-voltage pulsed current (HVPC) is the most common modality. Parameters such as ES dosage, polarity, and electrode placement is determined by trained professionals, as dictated by wound characteristics. Duration of treatment is usually 45 to 60 min, delivered 5 to 7 times per week. Many cell types show directional migration toward an external electric field, such as epithelial cells, fibroblast, lymphocytes, macrophages, endothelial neuronal cells, and stem cells; this process is known as galvanotaxis or electrotaxis [72]. If treating a wound with an infection present, the current selected should be a net positive charge in order to attract negatively charged neutrophils. If the goal is to facilitate the proliferative phase for angiogenesis and granulation tissue formation, a net negative charge would attract positively charged fibroblasts. Nonetheless, an RCT showed that overall ES improved blood flow and wound area reduction regardless of whether a wound was applied with a positive or negative charge [91].

## 20. Biological Modalities

Bioactive materials are those derived directly from human tissue sources, and include: cultured living allografts, human amnion/chorion membrane allografts, epidermal grafts, and autologous dermal matrices. *Cultured living allografts* are made up of epidermal cells, dermal cells, or both supported by a biodegradable matrix. Two common products include Apligraf and Dermagraft, which both contain living cells derived from neonatal foreskin. These are not recommended for use on infected wounds. Apligraf is a bilayered product consisting of a type I bovine collagen scaffold that contains living human dermal fibroblasts and an overlying cornified epidermal layer of living human keratinocytes. Dermagraft is composed of cryopreserved human fibroblasts on a polyglycan scaffold indicated for use in the treatment of full-thickness diabetic foot ulcers. Prior to application of either, wound bed preparation is mandatory; wounds are debrided, moisture balance maintained, and infection is monitored.

Cultured living allografts improve outcomes over the standard of care, while other products have not met this level of success [92].

*Human Amnion/Chorion Membrane Allografts (HACMs)* are harvested from human placenta or umbilical cord and are processed for use in wounds to promote healing. They contain either both amnion and chorion tissue or single-layer amnion alone [71]. HACMs are non-immunogenic, reduce inflammation and scar formation, contain relatively high levels of growth factors, and have been shown to stimulate cellular proliferation as well as promotion of mesenchymal stem cell migration [93].

*Epidermal* grafting is the application of negative pressure to the skin to create fluid-filled blisters (suction blistering), during which the lamina lucida of the skin is cleaved from the underlying layers [94]. This separates the epidermis from the dermis. The blisters are then cut and emptied, and the loose skin is transferred side by side to the non-healing wound. The procedure is painless and allows for the efficient transfer of multiple epidermal micrografts to the recipient wound bed [71].

*Autologous Dermal Matrix (ADMs)* are allograft scaffolds that are produced by harvesting human skin. The dermis is then processed to remove the epidermis and all cellular components but maintain ECM structure [71].

## 21. Other Modalities

*Therapeutic ultrasound* delivers energy through mechanical vibrations in the form of sound waves at frequencies (>20 kHz) above detection by the human ear [71]. Ultrasound affects tissue through thermal and non-thermal mechanisms, which are determined by physical properties such as the frequency or number of oscillations and the intensity or level of power. The thermal effects associated with greater tissue absorption of energy increase with higher frequencies.

High-frequency US is used in the 1 to 3 MHz range to promote soft-tissue injury and occasionally has been reported to facilitate wound healing. Low-frequency US (LFU) is the most common type of US device used in wound care today, as low frequencies penetrate deeper in tissue and are typically transmitted at frequencies of 20 to 120 kHz [95]. LFU has been shown to effectively debride necrotic tissue, eradicate some strains of bacteria from the wound, and facilitate the wound healing process through two mechanisms, cavitation and acoustic streaming [96,97]. Cavitation is the vibrational effect of US on micron-sized gas bubbles that form in the blood and lymph and tissue fluids. Periods of high and low pressure in treated tissue can cause the bubbles to increase and decrease in size. Unstable cavitation occurs when microbubbles significantly increase in size and violently implode. On implosion, the bubbles release a large amount of energy, which is destructive to bacteria while being essentially harmless to healthy tissue and cells. This allows preferential and rapid liquefaction of adherent necrotic fibrin (fibrinolysis) and the destruction of microorganisms on wound surfaces. Acoustic streaming alters cell membrane permeability and second messenger activity, resulting in increased production of growth factors, macrophage activity, and fibroblast proliferation and migration to create a collagen-rich connective tissue matrix [71].

*Hyperbaric Oxygen (HBO)* therapy is defined as “treatment during which a patient inhales 100% oxygen while enclosed in a pressurized chamber exceeding 1.4 atmospheres absolute (ATA)” [71].

Normal patients breathing at sea level will have an arterial pO2 of 100 mm Hg. The majority of this oxygen is bound to hemoglobin, with only a small percentage of oxygen physically dissolved in the plasma. This same patient breathing 100% oxygen at 3 ATA will have a marked increase in the amount of oxygen physically dissolved in the plasma. Plasma pO2 values in excess of 1500 to 1800 mm Hg are routinely achieved in hyperbaric patients, and tissue partial pressures can reach supraphysiological oxygen levels of 250 to 300 mm Hg, significantly above the normal 30 to 40 mm Hg when breathing 1 ATA [71]. This provides tissue hyperoxygenation and stimulation of angiogenesis. While it would seem HBO should facilitate pressure-injury healing, there is currently no evidence basis for its use. It is indicated for chronic osteomyelitis and compromised flaps and grafts, but its use is not supported for treatment of pressure injuries [71].

*Telemedicine* has proven to be beneficial in wound care. Data from the telemedicine CICAT network in France were analyzed from 2005–2015, and 5794 wound patients were included [91]. Investigators found that telemedicine in this network increased access to professional expertise in remote and rural settings and proved to be cost effective. In addition, results demonstrated that 75% of wounds treated with telemedicine improved or healed, there was a 72% reduction in the number of hospitalizations, and 56% reduction in ambulance transfers to wound healing centers [92]. Telemedicine may be beneficial in reducing in-person visits to wound specialists, as non-healing wounds require frequent assessments that can be conducted virtually [93].

## 22. Pain

Pain is often described as one of the worst aspects of living with chronic wounds. Consequences of unremitting pain include sleep disturbance, immobility, poor appetite, and depression [94]. In an international survey in 2018 of people with chronic wounds, over 60% of participants reported the experience of pain “quite often” and all the “time” [96] Pain from pressure injuries has been described as aching, stabbing, sharp, tender, and tiring, and appropriate analgesia is recommended [95]. Afferent input from wound nociceptors provides ongoing activation of primary afferent neurons to the dorsal horns of the spinal cord, which in turn activates secondary afferent fibers in the posterior columns of the spinal cord that conduct impulses to the thalamus and brainstem, where tertiary afferents convey impulses to the somatosensory cortex, causing the person to experience pain [82,96]. For persons without SCI, the perception of pain is typically accompanied by an increase in supraspinal inhibition that prevents untoward sympathetic and motor reflex activities. The person with SCI, however, may not experience pain as afferent signals are often, but not always, blocked at the level of injury. Nonetheless, afferent pain signals can activate uninhibited reflex sympathetic and motor pathways by increasing excitatory post-synaptic potentials (EPSPs) at preganglionic sympathetic fibers within the intermediolateral regions of the cord [32,33], or at the anterior horn cells, respectively [98]. In the first case, for persons with SCI above T6, nociceptive input from the wound can cause a massive sympathetic reflex below the level of SCI resulting in hypertensive crisis called autonomic dysreflexia (AD) that can result in stroke, seizures, organ failure, or death [32]. It is usually accompanied by a severe headache, bradycardia, and facial flushing, along with pallor, cold skin, and sweating in the lower part of the body. Often caused by bladder or bowel dysfunction, the initial treatment of AD is to sit the patient upright, remove any tight clothing or constrictive devices, monitor blood pressure and vitals, and consider pharmacological intervention. When caused by a pressure injury that will not immediately heal, chronic hypertensive suppression with an alpha-adrenergic antagonist will likely be required [32].

In the second case, the increased EPSPs at the alpha motor neurons below the level of SCI result in a velocity-dependent increase in muscle tone and stretch reflexes associated with hypertonia, termed spasticity [98,99]. Many people with SCI have stable spasticity managed by pharmacological agents such as gamma aminobutyric acid (GABA) agonists or alpha-adrenergic agonists, but a pressure injury may increase motor reflexes because of its afferent activation of EPSPs through spinal interneurons, and additional spasticity intervention may be required until the wound heals. Increased spasticity and associated spasms can be especially problematic due to the likelihood of inadvertent damage to the healing pressure injury.

Notably, it is important for the interdisciplinary team to be aware of the potential provocation of AD and spasticity during dressing changes and wound debridement; local analgesics administered prior to intervention may prevent or minimize these dangerous comorbidities [32].

## 23. Surgical Interventions

Surgical consultation should be provided for pressure injuries that have advancing cellulitis or that are a suspected source of sepsis, have undermining, tunneling, sinus tracts and/or extensive necrotic tissue that is not easily debrided conservatively, and for those stage 3 and 4 wounds that are not closing with conservative treatment [1]. The goals of surgical intervention include necrotic bone excision, defect filling with fascia and muscle, improved vascularity, and minimization of a bony prominence [1,2]. If the pressure injury has not lost tissue, it may be closed by primary intention (sutures, staples, glue), but this is seldom the case. Pressure injuries that lack only portions of the skin may be able to granulate closed if they are small. If large and/or recalcitrant to conservative management, the pressure injury may require a skin graft to speed the healing process. Grafts rely on the tissue to revascularize at its recipient site.

Wounds that have damaged tendon, muscle, or bone may require transplantation of these tissues with flaps of skin, muscle, fascia, and/or bone to fill the defects [100]. Flaps (as opposed to grafts) bring their vascular supply with them and are either locally transported to the wound or are from a distant site (free flap) [100]. Free flaps involve tissue that is freely removed from one area, detaching the nutrient artery and vein from their supply vessels and moving them to the recipient site, where these vessels are reattached to new supply vessels. Myofasciocutaneous flaps contain bulky muscle, deep fascia, and skin, and historically have been the best choice for managing recalcitrant pressure injuries, as they better tolerate pressure [101]. Wounds that are high risk for infection and have large dead space are ideal for muscle flaps [101]. Considerations include size and tissue components necessary for reconstruction, the resultant function and morbidity of the donor site, and the eventual function and aesthetic outcome of the recipient [101]. A 2018 study compared outcomes of fasciocutaneous flaps and gluteus myocutaneous flaps in patients with sacral pressure injuries and found that myocutaneous flaps had fewer episodes of partial necrosis (0 versus 32.5%), wound dehiscence (26.6 versus 47%), seroma formation (0 versus 17.6%), hematoma formation (0 versus 5.8%), and lower mean drainage time (5 versus 12 days); the overall rate of success was 86.6% for myocutaneous flaps compared with 58.8% for fasciocutaneous flaps [102] While local fasciocutaneous flaps may be an excellent option for smaller sacral lesions in patients with an adequate subcutaneous cushion, the gluteus maximus myocutaneous flap is one of the most reliable and safe flaps used in the management of sacral ulcers [102].

### Peri- and Post-Operative Care

A SCI Medicine consultant should be involved before and immediately after the surgical procedure to assist with the management of the many comorbidities associated with SCI [2]. The ERAS Society has provided a few strong recommendations based on “high” evidence regarding antibiotic prophylaxis and infection prevention; perioperative antibiotics should be given 1 h prior to surgery and continued for 24 h after surgery [103]. In the immediate post-operative period, vasopressors may be required to avoid hypotension as it has the potential to compromise flap blood supply; however, AD is also likely and needs to be monitored for and managed judiciously. Intravenous fluids can be used to improve hypotension, but excessive fluid administration should be avoided due to concerns of flap failure as well as local and systemic complications. Bladder management with an indwelling urethral catheter is essential to prevent wound soiling and to ensure adequate drainage, especially for those persons with SCI above T6 who are at risk of AD [2,104]. Similarly, a neurogenic bowel care program should be employed to minimize fecal soiling and likelihood of AD [2,105]. A specialty support surface should be provided in the immediate post-operative period and throughout recovery. Frequent reposition should be provided to avoid pressure on or disruption to the surgical site. Frequent flap checks are essential to ensure flap perfusion; flaps should be checked every 30 to 60 min for the first 2 to 4 h and then every 1 to 2 h for the additional 24 h following surgery. When the surgical site is sufficiently healed, a progressive sitting protocol should be employed [1,2].

## 24. Venous Thromboembolism Prophylaxis

Venous thromboembolism (VTE) prophylaxis with low-molecular-weight heparin (LMWH) is recommended for persons with chronic SCI who are re-hospitalized for medical illness and those undergoing surgical procedures [106]. The combination of relative immobility, systemic inflammation, and surgical intervention puts these persons at relatively high risk.

## 25. Psychosocial Factors

There are seven recommendations at A level clinical strength in prevention and treatment of pressure injuries listed in the EPUAP/NPIAP/PPPIA Clinical Practice Guideline, one of which describes the importance of providing education in pressure-injury prevention and treatment as part of a quality improvement plan to reduce the incidence of pressure injuries [1,107]. Clinicians may also benefit from increased wound education; studies report nurses desire more education and believe that their education is insufficient, and many physicians and residents were found to have low levels of pressure-injury knowledge [108].

### 25.1. Access to Specialized Care

Initiating early wound care can aid in timely wound-healing [3]; however, patients with pressure injuries are often unemployed, marginalized, and isolated. They commonly also incur additional out of pocket expenses for transportation, parking, telephone bills for medical follow-up, home health aide services, dressing supplies not covered by insurance, and drug costs if they have no prescription plan [109]. Financial barriers pose a challenge in accessing specialized care, and individual patient care costs may range from USD 20,900 to 151,700 according to the Agency for Healthcare Research and Quality (AHRQ) [110]. Lack of access to specialized wound care services can result in untimely care that has lethal implications.

### 25.2. Quality of Life

Pressure injuries significantly affect quality of life due to reduced physical activity, decreased independence, social isolation, pain, fear, and anxiety [8]. In a survey provided to people with chronic wounds, anxiety was the most common mental health disorder among 81.5% of those surveyed [111]. Studies report that 60% of people living with chronic wounds expressed higher-than-average anxiety [112]. People with chronic wounds tend to suffer higher rates of emotional distress and are less capable of coping with stressful events [113]. According to the Chronic Wound Quality of Life (CW-QoL) conceptual framework, the six key stressors encountered by people living with chronic wounds include: (1) wound status and treatment; (2) pain and other wound-related symptoms; (3) functional status (i.e., mobility, self-care, sleep); (4) emotions and psychological state (i.e., depression and anxiety); (5) financial resources and cost of care; and (6) social relationships (i.e., roles, citizenship, support) [114]. Stress activates the hypothalamic–pituitary–adrenal axis that produces vasopressin and cortisol, both of which impair wound healing [115]. In a study by Ebrecht et al., the impact of stress levels on wound healing was investigated. Patient stress levels were measured using the Perceived Stress Scale. Subjects exhibiting slow healing rated higher levels of stress during the study and presented higher cortisol levels 1 day after biopsy than the fast-healing group [115].

### 25.3. Social Isolation

Patients with chronic wounds commonly feel embarrassed about the smell and fluid leakage from wounds and, as a result, may intentionally avoid social contacts and activities. They often feel detached and emotionally distant from their families and friends, rendering it difficult to maintain meaningful relationships [111]. Those with SCI are further burdened with community barriers to wheelchair mobility, transportation limitations, and societal barriers to persons with disabilities.

## 26. Conclusions

In addition to motor paralysis and sensory loss, persons with SCI have multiple comorbidities, including neurogenic orthostatic hypotension, autonomic dysfunction, neurogenic restrictive and obstructive lung disease, neuropathic pain, spasticity, neurogenic bladder, neurogenic bowel, neurogenic obesity, sarcopenia, and metabolic syndrome (including diabetes mellitus, hypertension, dyslipidemia, and systemic inflammation) that increases their risk of pressure injuries. Early education about pressure-injury prevention, including appropriate support surfaces, wheelchair and seating systems, frequent repositioning, optimal transfer techniques, nutrition, physical activity, weight management, and smoking cessation may provide the best strategy for pressure-injury treatment in the form of prophylaxis [1,2]. Once a pressure injury occurs, early wound care can aid in timely wound-healing [3], but protein malnutrition can rapidly occur due to diminished lean mass/protein reservoirs related to the SCI. Management requires multidisciplinary intervention to determine and fix the cause of the pressure injury, optimize psychosocial support, provide complete pressure relief of the wound, optimize nutrition, minimize wound bioburden, optimize support surfaces and wheelchair seating systems, eradicate infections and if necessary, provide surgical intervention with myofasciocutaneous flaps and appropriate post-operative care.

## Figures and Tables

**Table 1:** Suggested support surface overlay or mattress selections for pressure-injury prevention and treatment based on Braden mobility and moisture subscale scores. AF—air fluidized; AMG—Australian Medical Grade; AP—alternating pressure; CLP—constant low pressure; LAL—low air loss.

	Braden Mobility Subscale Scores	
Braden Moisture Subscale Score	4 or 3 (No Limit or Slightly Limited)	2 or 1 (Very Limited or Immobile)
4 or 3 (Rarely or Occasionally Moist)	Reactive/CLP (air, foam, gel or viscous fluid, or combinations) AMG Sheepskin Overlay (Prevention Only)	Reactive/CLP Active with AP Feature
2 (Very Moist)	Reactive/CLP Reactive/CLP with LAL Feature	Reactive/CLP Active with LAL Feature
1 (Constantly Moist)	Reactive/CLP Reactive/CLP with LAL Feature	Reactive/CLP Active with LAL Feature Reactive/CLP Active with AF Feature (Treatment Only)

## Data Availability

Not applicable.
